# A new integrated hybrid technique for risk assessment in human-robot collaboration

**DOI:** 10.3389/frobt.2026.1916227

**Published:** 2026-09-09

**Authors:** Kristyna Hamrikova, Daniel Hartmann, Ales Vysocky, Vendula Laciok, Radim Hercik, Ales Bernatik

**Affiliations:** 1 Faculty of Safety Engineering, VSB – Technical University of Ostrava, Ostrava, Czechia; 2 Faculty of Mechanical Engineering, VSB –Technical University of Ostrava, Ostrava, Czechia; 3 Faculty of Electrical Engineering and Computer Science, VSB –Technical University of Ostrava, Ostrava, Czechia

**Keywords:** collaborative applications, collaborative robot (cobot), human–robot collaboration, occupational health and safety, risk assessment

## Abstract

Human–robot collaboration offers new opportunities to increase productivity, flexibility and ergonomics in work processes, however, it also introduces specific risks associated with a shared workspace between humans and robots, for which traditional risk assessment techniques may not be sufficiently effective. This article summarizes selected risk assessment techniques designed for the field of Human-Robot Collaboration (HRC) and evaluates their strengths and weaknesses with respect to their applicability in a collaborative environment. Based on the identified limitations, a new hybrid technique is presented that combines Hierarchical Task Analysis (HTA), Hazard and Operability Study (HAZOP), Process Failure Mode and Effects Analysis (HRC–PFMEA) and Risk matrix. The proposed approach integrates qualitative and quantitative risk assessments, enables risk identification at an early stage of system design, and increases the transparency of work processes. The applicability and logical workflow of the proposed method are demonstrated using a simulation scenario of an assisted assembly workstation created in a digital environment.

## 1 Introduction

The first section of the paper is usually an introduction section, in which the background, the topic and aims are described. The main results and principal conclusions should be highlighted in this section.

The novelty of this article lies in the proposal of a new integrated hybrid risk assessment technique tailored to human–robot collaboration. The proposed technique combines HTA, HAZOP, HRC–FMEA, and a risk matrix into a logically coherent framework applicable to HRC. This framework enables structured task decomposition, systematic identification of deviations, semi–quantitative risk analysis, and final risk evaluation within a single integrated procedure.

**FIGURE 1:**
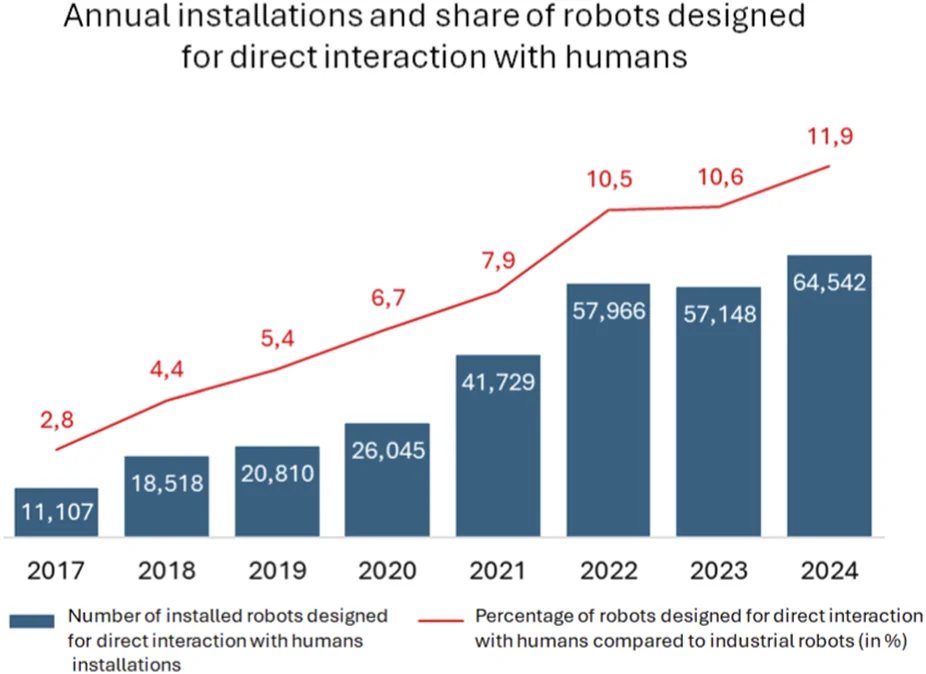
Annual installations and share of robotic systems designed for direct interaction with humans (Internation Al Federation of Robotics, 2025)

The field of human–robot collaboration (HRC) has undergone rapid development in recent years (see Figure 1) and is fundamentally transforming the world of work. With ongoing advances in robotic technologies and workplace safety systems, the scope of collaborative applications continues to expand. The primary objective of HRC is to achieve safe and efficient cooperation between humans and robots. Unlike conventional industrial robots, which typically operate in isolated environments, robots intended for collaborative applications are designed to share a workspace with human operators. Owing to advanced sensors, sophisticated control architectures, and integrated safety functions, such systems are designed to reduce the risk of operator injury (Weiss et al., 2021). This symbiotic interaction enables the combination of machine precision and strength with human flexibility and decision-making capabilities (Rahman et al., 2024). However, full automation, defined as the complete exclusion of human operators from production processes, as promoted in the context of Industry 4.0, cannot be applied universally, as technical and economic constraints may arise (Kolbeinsson et al., 2019). It is therefore necessary for humans to remain involved in production systems and work alongside intelligent machines, including robots designed for human collaboration (Weiss et al., 2011; Gualtieri et al., 2020). At the same time, this development introduces a number of challenges, as the integration of collaborative applications into assembly lines is more complex than the automation of individual work processes (Kolbeinsson et al., 2019), particularly from a safety perspective. In HRC environments, safety cannot be ensured solely by restricting access to hazardous areas to prevent human approach to, or contact with, the machine.

One of the barriers to the adoption of HRC is the proper establishment of workplace safety, not only from the perspective of employees working directly with the robot, but also with regard to worker safety during maintenance and service operations throughout the robot lifecycle. Ensuring overall workplace safety and protection within the risk assessment process in collaborative robotics therefore represents a significant challenge (Keshvarparast et al., 2024; Cardoso et al., 2021). This view is further supported by studies (Jocelyn et al., 2023; Villani et al., 2018; Hentout et al., 2019), which emphasise that safety in human–robot interaction (HRI) remains a critical issue.

In line with the concept of harmonious human–machine collaboration emphasised by Industry 5.0 (Almusaed and Yitmen, 2024), it is necessary to assess both physical and psychological risks already at the design stage of a collaborative workplace. A safe and inclusive working environment suitable for a diverse workforce must be ensured (Keshvarparast et al., 2024). Integrating risk assessment directly into the early virtual simulation phase supports this design-stage evaluation while delivering a major economic benefit: modifying a digital model in a simulation incurs minimal costs compared to the expensive physical retrofits required if hazards are discovered only during actual workplace installation. The authors of studies (Keshvarparast et al., 2024) and (Lorenzini et al., 2023) also stress the need to extend this perspective to psychological and cognitive aspects, which have a substantial influence on both the safety and acceptability of working with robots.

### 1.1 Dangerous situations in collaborative applications

The first example is an experiment conducted by the German Aerospace Center (DLR) a programme of the Institute of Robotics and Mechatronics (Are You Still Doubting Collaborative, 2024), which has maintained long-term cooperation with KUKA. KUKA, whose name is derived from the initials of its founders, Keller und Knappich Augsburg, is a leading German supplier of intelligent automation solutions. The experiment was carried out primarily using the KUKA LBR (“Leichtbauroboter,” lightweight robot) iiwa (“intelligent industrial work assistant”). DLR performed a series of controlled experiments aimed at determining the forces generated during collisions between the robot and a human and at assessing the resulting injuries.

The researchers intentionally simulated several situations that may arise in industrial practice:

- direct contact between the robot and a human arm,
- scenarios involving rapid motion of the end-effector,
- scenarios involving the use of sharp tools (e.g., a screwdriver, knife, or needle).

The robots operated in two modes: without active collision detection and with active force detection combined with automatic stopping. The tests were conducted on soft biomaterials (e.g., lamb tissue and artificial skin), and in some experiments, the researchers also examined direct contact with a human arm under controlled conditions.

The experimental results showed that, in the mode without collision detection, deep penetration of soft tissue occurred, including cut and puncture injuries. After activation of the force and speed limiting function, the contact forces were significantly reduced and no injuries were observed. DLR concluded that robots intended for HRC can be considered safe only if:

- are equipped with integrated force and torque sensors,
- operate with limited speed and kinetic energy (in accordance with ISO 10218-2:2025),
- and are used with appropriate tools that do not have sharp edges.

The researchers emphasized that robots intended for HRC can be considered safe only when operated under controlled conditions with limited forces and speeds. Their safety is therefore not inherent, but rather depends on their design, configuration, and manner of use.

In addition, DLR issued a 2020 advisory for customers using UR (Universal Robots) systems distributed by Tech Con, highlighting two issues affecting the UR3, UR5, and UR10 models.

These issues were:

1. Overvoltage at the output of the control cabinet power supply,
2. Unexpected motion following an encoder error.

These faults may lead to hazardous situations, such as fire or worker injury caused by unexpected movement. According to the authors, the purpose of the report was to provide an advance warning of a forthcoming product update (Are You Still Doubting Collaborative, 2024).

The first real-world example of a serious accident is the incident that occurred in Moscow during a chess tournament. During a game with a UR robot, a seven-year-old child suffered a broken finger. The cause was reportedly a failure in system integration and an inadequate risk assessment of the collaborative application, combined with the use of a rigid end-effector and the absence of safety sensors for human presence detection. The system was not designed to account for the child’s unpredictable behaviour. Tech Con, the manufacturer of UR robots, stated that the robot was equipped with the PFL (Power Force Limiting) mode and should have stopped upon contact; therefore, the incident should not have occurred. However, this would only have been the case if an appropriate risk assessment had been carried out, a soft end-effector had been used, and safe proximity detection had been ensured, for example, by means of a high-speed camera. None of these conditions were met in this case.

Study (Sanders et al., 2024) analysed 77 robot-related accidents based on Occupational Safety and Health Administration (OSHA) Severe Injury Reports from 2015 to 2022. The study reached the following conclusions. A total of 77 accidents were recorded, of which 54 involved stationary robots and 23 involved mobile robots. The most common injuries associated with stationary robots were finger amputations, followed by fractures, lacerations, contusions, burns, and electric shocks. Thirty-two injuries (48%) involved the fingers and hands, 19 injuries (29%) affected the head, torso, and pelvis, and 13 injuries (20%) involved the arms, legs, and feet. In the case of mobile robots, the most common injuries were fractures of the legs and pelvis. Specifically, these cases included 9 leg fractures, 3 pelvic fractures, 3 foot fractures, 3 cases of torso soreness, 2 hand fractures, 1 finger amputation, 1 leg avulsion, 1 pelvic contusion, 1 crushing injury to the torso, 1 laceration of the arm and leg, and 1 case of leg soreness. Recurring hazardous situations included unexpected robot movements, for example, during maintenance or adjustment, and human entry into the path of a mobile robot, resulting in foot run-overs, leg fractures, or pelvic fractures. The main identified causes included inadequate or easily bypassed guards and interlocks, failure of Lockout/Tagout (LOTO) measures during maintenance, insufficient collision detection systems that were unable to detect individual limbs, inadequate warnings, and, in some cases, untrained or inexperienced workers, as well as failure to follow established procedures (Winner et al., 1998).

## 2 Related works

Research indicates that traditional approaches to risk analysis are not always sufficient for collaborative robotics. Standardized procedures, such as ISO 12100:2010 and ISO 10218-1/-2:2025, provide a fundamental framework; however, their application in the dynamic environment of shared workspaces remains limited (Gualtieri et al., 2020; Cardoso et al., 2021).

Considerable attention is devoted to the ergonomics of human–robot collaboration. The literature (Gualtieri et al., 2020; Lorenzini et al., 2023) shows that poorly designed workplaces may lead to excessive physical strain, such as repetitive upper-limb movements and static or awkward body postures, as well as psychological stress, for example, stress induced by robot motion. Studies (Gualtieri et al., 2020; Sanders et al., 2024) recommend applying the principles of concurrent engineering during the design process in order to simultaneously consider product parameters, the manufacturing process, workplace layout, and the psychophysiological needs of workers. Cognitive ergonomics also plays an important role, particularly in relation to intuitive robot behaviour, including natural motion trajectories, predictability of actions, and visual or auditory feedback (Gualtieri et al., 2020; Lorenzini et al., 2023).

With the increasing mobility and autonomy of robots, it is essential to ensure that they do not pose a hazard to people working in the same space. To support human wellbeing and improve overall system productivity, a range of strategies in management, safety, and worker support must be implemented (Ai et al., 2019).

An important finding is that, while mechanical safety is already relatively well addressed by international standards and existing research (Gualtieri et al., 2020; Cardoso et al., 2021), process safety, as well as psychological and cognitive aspects, remain largely at the level of experimental studies, and no unified methodology has yet been established for these areas (Keshvarparast et al., 2024; Lorenzini et al., 2023). This imbalance indicates the need for further research to achieve a better balance between physical and psychological worker safety (Keshvarparast et al., 2024).

The above-mentioned studies show that the implementation of HRC robots requires a holistic approach combining technical standards, safety measures, ergonomics, and organisational factors. Only through the integration of multiple approaches can workplaces be created that are safe, efficient, and acceptable for a diverse workforce.

The ISO 10218-2:2025 standard explicitly states:

“The hazards associated with the use of robots are well known; however, the sources of these hazards are often specific to individual robotic applications. The number and types of hazards are directly related to the nature of the automation process and the complexity of the application. The risks arising from these hazards vary depending on the robot used, its safety features, and its integration, installation, programming, operation, and maintenance. Given the wide range of robotic applications and robotic cells, it is not possible to provide an exhaustive list of all significant hazards, hazardous situations, or hazardous events associated with robotic applications. Moreover, the same type of application may involve different levels of risk due to differences in design related to its intended use (e.g., spray painting of large or small parts, handling small hazardous loads such as hot metal bolts, or large non-hazardous loads such as boxes of paper tissues).” (International Organization for Standardization, 2025a).

This suggests that applications of the same nature may present different levels of risk due to differences in design tailored to their intended purpose. It also illustrates that risk assessment must be carried out within a specific context, taking into account not only the type of robot, but also the operating environment, system integration, and intended use. Consequently, it is not possible to establish a standardized list of risks, which necessitates a systematic analysis for each new robotic application.

The newly introduced Annex G in ISO 10218-1:2025 provides a detailed description of the “Methods for verifying and validating design requirements and risk reduction measures.” This annex identifies all clauses of the standard to which the verification and validation methodology applies. These methods include visual inspection, practical testing, measurement, or risk assessment and/or FMEA evaluation. It should be noted that, apart from FMEA, the standard does not specify any particular techniques (International Organization for Standardization, 2025b).

The technical specification ISO/TS 15066:2016 refers to the term “collaborative robot.” However, the revised ISO 10218-2:2025 standard, which incorporates this earlier specification, emphasizes that the term “collaborative robot” has been removed and is no longer used. It is now stated that only an application, once verified and validated, may be considered collaborative (International Organization for Standardization, 2025a). The authors of this article regard this change as beneficial for the conformity assessment of HRC robots.

The term “collaborative application” refers to an application that includes one or more collaborative tasks. An “application” denotes the intended use and purpose of a robot or robotic system, that is, the process or task(s) to be performed. A “collaborative task” is part of a robotic sequence during which both the robotic application and the operator are present within the same safeguarded space (International Organization for Standardization, 2025a). Determining whether an application is collaborative involves, at a minimum, establishing whether the application is sufficiently safe for people within the robot workspace. Therefore, a risk assessment of the application must be carried out. This process helps determine the measures required for risk reduction (Jocelyn et al., 2023).

Based on this determination, the integrator may carry out the conformity assessment of the robot. If the robot has not been integrated into its operating environment, it is still classified as partly completed machinery under the applicable EU legislation.

ISO 10218:2025 provides guidance on the implementation of three safety modes that enable collaboration between humans and robots. These modes are: [1] hand guiding control (HGC), [2] speed and separation monitoring (SSM), and [3] power and force limiting (PFL) (International Organization for Standardization, 2025a). These three modes may be applied individually or in combination (Jocelyn et al., 2023). Previously, ISO 10218:2011 defined four modes for collaborative applications, with the fourth being Safety-Rated Monitored Stop (SRMS). In ISO 10218:2025, however, this feature is no longer classified as a collaborative safety mode, but only as one of the robot’s safety functions.

Conducting a thorough risk analysis and assessment is also essential in the field of HRC in order to determine the necessary protective measures and safeguards required to mitigate the identified risks. Furthermore, there is still no structured framework for aligning the design of protective measures with the results of risk analysis and assessment (Giallanza et al., 2024).

Study (Huck et al., 2021) argues that the risk assessment and mitigation procedures currently proposed by normative standards are based primarily on expert knowledge supported by intuitive techniques, such as brainstorming, and simple tools, such as checklists. Although this approach is generally accepted, its application to HRC systems may be challenging due to their complexity, limited practical experience, and the difficulty of predicting human behaviour. In recent years, several new approaches to risk analysis and assessment have been proposed to address HRC. These include, for example, task-oriented techniques, formal verification methods, and simulation-based approaches. However, the authors emphasize that a substantial gap still exists between research and industrial practice (Giallanza et al., 2024).

According to (Chemweno et al., 2020), more specific guidance is needed for conducting risk analysis and risk evaluation in collaborative environments, with particular emphasis on the human factor. In addition, there is still no structured framework for aligning the design of protective measures with the outcomes of risk analysis and evaluation.

Study (Hanna et al., 2022) also highlights shortcomings in the approaches currently used for risk analysis and assessment. In particular, it criticises the fact that current regulations focus primarily on the design of protective measures, while paying only limited attention to the principles of active safety.

The increasing level of automation and digitalisation exposes industrial sectors to the threat of cyberattacks, which may result in data theft, product damage, and injury to personnel (Giallanza et al., 2024).

Consequently, appropriate guidelines and protocols need to be developed to prevent risks related to networking and cyberattacks and to incorporate both safety and security considerations into the design of collaborative systems (Giallanza et al., 2024).

In response to these concerns, experts and several studies emphasize two key points: first, the importance of risk assessment, and second, the need to consider all possible risks. According to the European Commission guidelines, companies should identify risks arising not only from physical factors, but also from psychological, organisational, communication-related, cultural, and systemic factors. Similarly, according to the International Organization for Standardization, robot risk assessments should include all reasonably foreseeable risks that could result in worker injury (Lee et al., 2021).

The authors of study (Khalid et al., 2018) propose a two-stage safety methodology aimed at improving data security in key interconnected nodes and thereby mitigating cyberattacks through an intelligent module for real-time system security monitoring. For future development, they recommend the implementation and further enhancement of Internet Protocol Security (IPsec) and the development of appropriate design guidelines to ensure the safety and security of complex multi-stage collaborative cyber-physical systems (CPS).

### 2.1 Safety engineering techniques designed for human–robot collaboration

One of the techniques proposed for use in the field of HRC is the modified Hazard and Operability Study–Unified Modelling Language (HAZOP–UML) approach, which is considered more broadly applicable to HRC due to the use of UML diagrams. Its purpose is to identify hazards arising from robotic tasks and their interactions with humans (Huck et al., 2021; Guiochet, 2016). Examples of the application of this technique can be found in studies (Martin-Guillerez et al., 2010; Hoang et al., 2012) and (Inam et al., 2018), where it was used in different case studies. These studies indicate that the technique is highly relevant for HRC applications; however, its practical implementation is demanding due to the need for UML–based modelling.

The System–Theoretic Process Analysis (STPA) technique is based on a hierarchical model of the system control structure that describes the interactions among individual subsystems, humans, and the environment. The method builds on the STAMP concept, which views safety as a problem of control and regulation within a sociotechnical system (Bensaci et al., 2019). Through a systematic procedure that includes the identification of hazardous control actions (Huck et al., 2021), STPA enables the analysis not only of failures in individual components, but also of risks arising from their interactions. The theoretical foundations of STPA are summarized in study (Leveson, 2012), which highlights the method’s advantage in integrating the human factor and software within a single analysis, as well as its relatively straightforward integration into systems engineering. Study (Adriaensen et al., 2021) confirmed the suitability of the method for collaborative robotics and its ability to capture human–robot interactions. The authors noted that STPA enabled a better understanding of the behaviour of the system’s control structure. In (Skokan and Vanzura, 2022), the method demonstrated its ability to link human and technical aspects of the system and to identify systemic factors affecting safety. In study (Zacharaki et al., 2021), STPA was extended to include real-time decision-making, and it was shown that the technique can also be applied during the operational phase of the system to support transitions between hazardous and safe states. The findings of these studies indicate that STPA is a flexible tool suitable for complex applications in collaborative robotics and autonomous systems.

RiskSOAP is an innovative methodology based on the STPA technique that focuses on situational awareness, which it considers a key safety factor, making it highly suitable for HRC (Huck et al., 2021). The development and application of this technique are presented in study (Chatzimichailidou and Dokas, 2015).

Another adaptation of traditional analytical techniques for HRC was introduced in study (Antonelli and Stadnicka, 2019). In this study, Failure Mode and Effects Analysis (FMEA) was adapted to identify potential hazards arising from human or robot errors in collaborative assembly processes, resulting in the modified Human–Robot Collaboration–Process Failure Mode and Effects Analysis (HRC–PFMEA) technique (Huck et al., 2021).

Another technique that can be applied in HRC is Fault Tree Analysis (FTA). In study (Yakymets et al., 2014), this technique was enhanced by the automatic generation of fault trees and by qualitative and quantitative analysis within the RobotML modelling environment. Fault trees and the related analyses can therefore be generated automatically, although they may still also be performed manually.

The final approach discussed for the field of HRC is Hierarchical Task Analysis (HTA). HTA is a structured and objective approach used to describe user performance during task execution. This and other task-oriented approaches to human–robot collaboration are also supported by study (Marvel et al., 2014). In its most basic form, HTA provides an understanding of the tasks that users must perform in order to achieve specific goals (Hornsby, 2010). Beyond HRC, this technique has been widely applied in various fields, including surgery, ergonomics, forestry, and industrial domains, where it is used for the allocation of tasks within complex systems (Wang et al., 2023), as well as for error prediction (Stanton, 2006). HTA was also identified in study (Antonelli and Stadnicka, 2019) as a useful approach for the design of collaborative applications, and it is further presented in study (Tan et al., 2010), which focuses on planning human–robot collaboration. In that study, the authors used HTA to decompose the overall task and subsequently derive specific hazardous situations from the individual task steps. They then applied a semi-quantitative assessment based on selected parameters.

### 2.2 Limitations of the mentioned techniques

As with the conventional HAZOP technique, one of the main limitations of HAZOP–UML is that its detailed analysis and associated time demands generate a large number of possible deviations. This technique does not cover all types of risks arising from complex human–robot interactions and is not universally applicable across the entire system design. Another limitation is its strong reliance on the analyst’s expert judgement and on the correctness of UML modelling. If the models are not accurate and complete, the outputs of HAZOP–UML may be incomplete or misleading, since all identified deviations are derived from them. Quantitative risk assessment is also difficult due to the lack of data on software failure rates and human errors (Guiochet, 2016; Martin-Guillerez et al., 2010; Hoang et al., 2012). These limitations are also acknowledged in studies (Guiochet, 2016; Martin-Guillerez et al., 2010) and (Hoang et al., 2012), which refer to combinatorial complexity, dependence on expert judgement, and the need to complement this method with additional techniques, such as Preliminary Hazard Analysis (PHA) or Fault Tree Analysis (FTA). The CASE tool developed by the authors of study (Martin-Guillerez et al., 2010) only allows export in CSV format, which limits its broader applicability for generating output artefacts such as risk lists, tables, or recommendations. Study (Inam et al., 2018) applied the HAZOP technique only in the form of a manual analysis, without the use of UML diagrams or tool support, while referring only generally to HAZOP–UML. At the same time, the authors acknowledge the general limitation of classical HAZOP in that it is not capable of identifying all possible risks.

Among the most frequently cited limitations of the STPA technique are its high demands in terms of time and data, its reliance on the analyst’s experience, the lack of quantitative evaluation, limited tool support, and its difficult applicability to large-scale systems. Study (Adriaensen et al., 2021) points out that the full application of this method is highly extensive and requires a substantial amount of input data and modelling. The authors noted that it was necessary to limit the analysis to only part of the system, since analysing the entire structure would have been excessively complex and time-consuming. The authors of studies (Adriaensen et al., 2021) and (Skokan and Vanzura, 2022) further state that the interpretation of results and the correct identification of causes depend heavily on the assessor’s experience and expertise. This may lead to differing results across teams or experts. STPA provides a qualitative framework, but it does not allow precise quantification of the probability or severity of individual risks. This limitation is also noted in study (Leveson, 2012), which states that the technique is more suitable for systematic hazard identification than for numerical risk evaluation. According to (Skokan and Vanzura, 2022) and (Zacharaki et al., 2021), there is still a lack of methodology or software capable of fully automating the analysis and ensuring the reproducibility of results. Manual assignment of system states and control actions increases the likelihood of error and reduces efficiency. Studies (Adriaensen et al., 2021) and (Zacharaki et al., 2021) also emphasize the difficulty of applying the method to large systems. As the number of components and interactions increases, the analysis becomes less transparent, leading to a so–called combinatorial explosion similar to that observed in HAZOP-based techniques.

The main limitation of the PFMEA technique lies in the limited transferability of traditional FMEA evaluation criteria. The authors note that the standard FMEA approach is not directly applicable to collaborative systems, because the historical data required to estimate the probability and severity of failures are not available. They therefore developed modified evaluation scales for severity, occurrence, and detection. Following these modifications, the risk assessment relies more on qualitative estimates based on a 1–10 scale than on real operational data, which increases the likelihood of differing results among assessors. The authors themselves also acknowledge the lack of practical validation under real operating conditions, since the case study was conducted in a laboratory environment (Antonelli and Stadnicka, 2019).

A broader limitation in this area is that the process of risk assessment and risk reduction recommended by current standards is still largely based on experience and expert knowledge, supported by intuitive methods such as brainstorming and simple tools such as checklists. Although this approach is generally accepted, its application to HRC systems may be challenging (Huck et al., 2021). The discussion in (Antonelli and Stadnicka, 2019) further suggests that HRC lacks standardized frameworks for evaluating severity, occurrence, and detection, which limits the broader applicability of such techniques.

## 3 Materials and methodology

**FIGURE 2:**
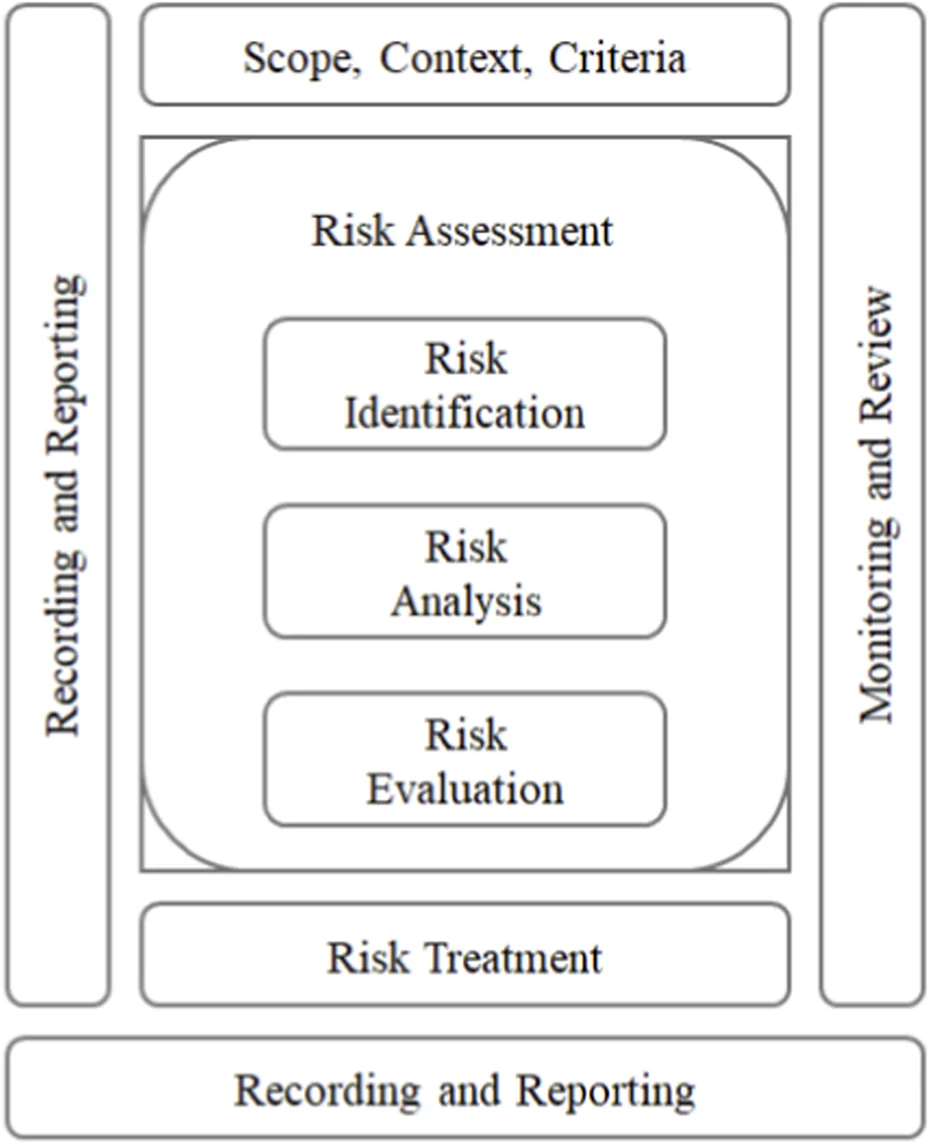
Risk management process diagram (International Organization for Standardization, 2018)

The terminology and procedural steps of the newly proposed technique are based on the ISO 31000:2018 risk management standard. This standard describes the process in general terms (see Figure 2).

Risk assessment is generally understood as a process involving the identification, analysis, and evaluation of the risks associated with adverse events.

Risk identification is the process of searching for, recognising, and describing risks. It involves identifying risk sources, events, their causes, and their potential consequences. This process may draw on historical data, theoretical analysis, expert judgement, and the needs and perspectives of relevant stakeholders (International Organization for Standardization, 2018).

Risk analysis is the process of developing an understanding of risk, including its nature and level. It involves a detailed examination of uncertainties, risk sources, consequences, likelihood of occurrence, events, scenarios, existing measures, and their effectiveness. Analytical techniques such as those described in IEC 31010:2019 are also applied at this stage of risk management. Risk analysis provides input for risk evaluation, for decisions on whether and how risks should be treated, and for determining the most appropriate strategies and techniques for managing them (International Organization for Standardization, 2018).

The purpose of risk evaluation is to support decisions regarding the acceptability or unacceptability of risks. Risk evaluation involves comparing the results of risk analysis with established risk criteria, such as those set by legislation, in order to determine whether further action is required (International Organization for Standardization, 2018).

The newly proposed technique for the risk assessment of collaborative applications seeks to address these shortcomings by using HTA and HAZOP for risk identification. The risk analysis stage is based on the individual steps of the HRC–PFMEA technique. Although one of the limitations of HRC–PFMEA is the lack of historical data and the resulting need for estimated values, the proposed methodology defines parameters to support their appropriate determination. The risk evaluation stage is then carried out using a risk matrix.

**FIGURE 3:**
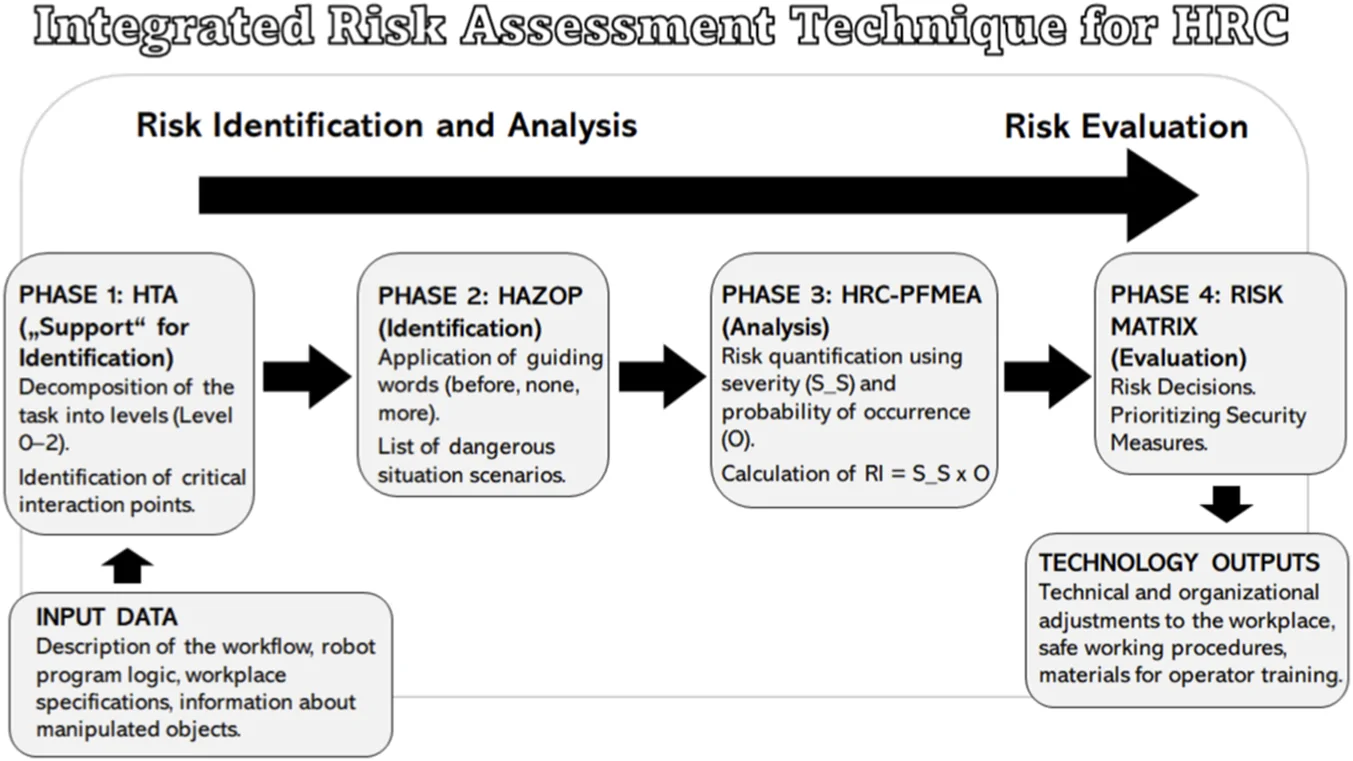
Logic of the proposed technique.

The logic of the proposed technique is illustrated in Figure 3.

The identification techniques used in the proposed technique are as follows:

- HTA–a structured decomposition of the work process into individual steps and human–robot interaction points.
- HAZOP–a systematic identification of deviations from the intended course of action for each HTA step.

The analysis technique used in the proposed technique is:

- Modified HRC–PFMEA–risk analysis based on the parameters Severity and Occurrence, including the calculation of the Risk Priority Number (RPN).

The evaluation technique used in the proposed technique is:

Risk matrix–used to determine risk acceptability and to prioritise corrective measures.

## 4 Methodology for selecting selected techniques

The selection of techniques used in the proposed hybrid technique was based on a review of the risk assessment techniques listed in IEC 31010:2019. In this study, IEC 31010:2019 served as the initial reference set containing a broad range of techniques. However, the standard does not specify which of these techniques are most suitable for the field of HRC. For this reason, the individual techniques were not adopted directly but were instead assessed in terms of their applicability to this specific domain.

The selection process took into account the specific nature of risk assessment in HRC. Risks in collaborative applications do not arise solely from technical failures but are also related to the structure of the work task, the sequence of activities, the shared workspace, human–robot coordination, the timing of individual actions, and the organisational conditions of the workplace. The objective was therefore not to identify a single universally applicable technique, but rather to define a set of complementary techniques covering the phases of risk identification, analysis, and evaluation.

In assessing suitable techniques, particular attention was paid to the following criteria: relevance to HRC, the ability to capture the structure of the work task and the sequence of activities, the ability to identify deviations and hazardous situations, the possibility of analysing causes and consequences, prioritize risks, and practical applicability under design-stage conditions. In addition, the transparency of the procedure and its consistency with the general framework defined by ISO 31000:2018 and ISO 12100:2010 were taken into account.

The analysis showed that no single technique provides sufficient coverage of all required phases of risk assessment in HRC. Some techniques are suitable for structuring the task, others for identifying deviations, others for analysing causes and consequences, and others for the final evaluation of risk significance. The proposed technique was therefore designed as a combination of methods with distinct yet complementary functions.

The HTA technique was selected for the risk identification phase. Its main contribution lies in the structured decomposition of the work process into hierarchically organised steps and in the definition of interaction points between humans and robots. In an HRC environment, such decomposition is essential because risk is closely linked to specific task steps, entry into the shared workspace, object handover, mode changes, and other interaction events. HTA does not itself perform risk analysis but provides the process structure required for the subsequent identification of hazardous situations. At the same time, its outputs can also be used in the specification of work procedures and related organisational measures.

HAZOP was selected as the subsequent identification technique. Its function is to identify deviations from the intended execution of individual task steps. In the proposed technique, HAZOP is applied to the critical steps identified through HTA. This makes it possible to systematically formulate deviation scenarios, their possible causes, and their potential consequences. This approach is particularly relevant for HRC, because many hazardous situations are associated with incorrect sequencing, premature or delayed actions, incomplete execution of a step, or inadequate coordination between humans and robots, rather than solely with conventional component failures.

The HRC–PFMEA technique was selected for the risk analysis phase. Its purpose is to support the quantification of the identified hazardous scenarios and to determine risk priorities. Compared with conventional FMEA, this modified form is better suited to the process-oriented nature of collaborative applications, as it allows technical failures, human errors, coordination failures, and communication deficiencies to be taken into account. At the same time, it preserves the link between the task step, the identified deviation, and the resulting risk assessment item. Given the limited availability of robust historical data for HRC, the Occurrence parameter in the proposed technique is treated as an expert estimate of the likelihood of occurrence, while the Detection parameter is retained as optional and is not quantified in the laboratory demonstration presented in this article. This adjustment reduces the risk of overstating the precision of the assessment under conditions of limited data availability.

A risk matrix was selected for the risk evaluation phase. Its function is to support the final classification of risks according to their significance and to establish priorities for the design of risk reduction measures. In the proposed technique, the matrix is not used for identification or analysis, but rather for evaluation and decision support. Its main advantage lies in the clarity of interpretation and its practical usefulness for communicating results to integrators, safety engineers, and other relevant stakeholders.

Other approaches reported in the literature for HRC were also considered during the selection process, particularly STPA, HAZOP–UML, and FTA. In the technical literature, STPA is regarded as a methodologically strong approach because it enables the capture of system interdependencies, control structures, and interactions between the technical and human elements of the system. However, its broader practical application is limited by higher demands in terms of system modelling, the scope of input information, the expertise of the assessor, and the time required for the analysis. Similar limitations also apply to HAZOP–UML, whose application depends on the development of a sufficiently detailed UML model and is associated with increased methodological and time demands. For these reasons, neither STPA nor HAZOP–UML was selected as the main basis of the proposed technique.

FTA was also considered, but it was not selected as the primary structuring technique. Its main limitation lies in its deductive principle, in which the analysis starts from a predefined top event and subsequently traces its causes. In the HRC environment, however, hazardous situations are highly application-specific and arise from various combinations of technical, human, and organisational factors. In the early design phase of a collaborative application, it may therefore be difficult to define a single representative top event that would adequately cover the full range of relevant scenarios. For this reason, FTA was not considered the most suitable primary technique for the initial structuring of risks in this study, although it may still be useful for the subsequent analysis of selected specific events.

The resulting technique is therefore not based on an attempt to identify a single universal method, but rather on a combination of techniques that reflects the sociotechnical nature of HRC. The selected set consisting of HTA, HAZOP, HRC–PFMEA, and the risk matrix establishes a coherent framework linking task structuring, deviation identification, cause-and-effect analysis, and the final evaluation of risk significance. The selection of this combination was guided by the need for methodological continuity, practical applicability, and usability in the early stages of collaborative application design.

## 5 Identification, analysis and evaluation of risk

This section presents the techniques used in the proposed hybrid technique. This chapter serves to understand the individual techniques and steps.

### 5.1 Risk identification techniques

Hierarchical Task Analysis (HTA) is a technique used primarily in ergonomics and human–machine systems to analyse tasks performed by humans (or systems). Its purpose is to decompose a complex task into smaller hierarchically organised steps (subtasks) and to identify where errors or safety risks may occur.

The technique focuses on what the human does, why it is done, and in what sequence.

The input data for HTA consist of a detailed description of the work process, ideally including the actual observed procedure performed by the operator, work instructions, a description of the workplace, and the allocation of human–robot roles, as well as the robot program (modes, logic, safety functions) and information about the manipulated object.

The objectives of HTA are as follows:

- to understand the activities of the operator and the robot within a specific process,
- to identify critical interaction points (shared workspace, object handover, entry into the robot workspace, mode change) that may lead to errors or risks,
- to create a structure to which the HAZOP technique can be systematically applied,
- to provide a basis for safe work procedures and training materials.

Task decomposition is typically structured as follows:

- Level 0: the overall goal (for example, the complete assembly operation),
- Level 1: sub-goals (process phases),
- Level 2: specific steps (individual human and robot actions).

Part of the output is the so-called plan, which defines the sequence or conditions under which the individual sub-steps are performed. It describes the order of steps and the decision points within the process.

Sub-steps of the procedure:

1. Define the overall goal–clearly specify the final objective of the activity (e.g., assembly of a component, transfer of an object, etc.).
2. Collect task-related information–observe the operator, review video recordings, consult experts, and examine work instructions and operating procedures.
3. Decompose the task into sub-steps–identify all activities necessary to achieve the goal.
4. Establish the hierarchy (task tree) – organise the steps into a hierarchical structure according to dependencies and the sequence of activities.
5. Define the plan–describe the sequence of tasks, conditions, and decision points (e.g., steps 2.1–2.3 may be performed in any order, followed by step 3).
6. Verify the hierarchy and plan with operators and experts–confirm that the decomposition is correct and reflects the actual work process.
7. Identify hazardous or error-prone steps–determine the critical interaction points where incorrect procedures, delays, inappropriate reactions, or improper interactions may occur.
8. Apply the HAZOP technique.

Selection of critical steps for targeted HAZOP application:

To ensure that the identification of deviations does not become a purely formal exercise based on mechanically applying all guide words to every step, detailed HAZOP analysis is prioritised for critical HTA steps defined by at least one of the following criteria:

- shared human–robot workspace,
- work in close proximity to the gripper/end-effector,
- presence of pinch points, entanglement, or crushing hazards,
- a step dependent on timing or signalling between the human and the robot,
- non-routine modes (setup, maintenance, troubleshooting).

The outputs of HTA include the hierarchical task structure, the plan, and a list of interaction points and critical steps.

Hazard and Operability Study (HAZOP) is a systematic technique used to identify risks, hazardous conditions, and operational problems in complex processes and systems. It is carried out as a team-based expert analysis in which guide words, presented in Table 1, are used to systematically identify possible deviations from the intended process, together with their potential causes and consequences. The objective is to identify critical deviations, hazardous scenarios, and their associated causes and consequences.

**TABLE 1:** Guidewords of HAZOP technique.

Guidewords	Significance
None	Complete negation of the project intention. A situation where an action or condition does not occur, even though it should have
More	Quantitative increase. A situation where an action or condition is excessive (greater than expected)
Less	Quantitative decline. A situation where an action or condition is insufficient (less than expected)
Reverse	The logical opposite of the intended design is achieved. A situation in which an action or state occurs in the opposite direction to that intended
In addition	All design plans are implemented together with accessories. A situation in which an unexpected event or condition occurs simultaneously with an expected one
Part of	The intention of the design is only partially achieved. A situation in which an action or state is incomplete
Other than	Complete replacement, where no original intention is achieved, but something completely different happens A situation in which a different state or action occurs than expected
Early	Something happens earlier than expected in terms of time (hours)
Late	Something happens later than expected due to the time (hours)
Before	Something happens earlier than expected in relation to order or sequence
After	Something happens later than expected in relation to order or sequence

In the proposed technique, HAZOP is applied to the individual critical steps identified by HTA, thereby extending the structured process description with scenarios describing what may go wrong at the technical, human, and human–robot coordination levels.

For each relevant HTA step, the following are defined:

- the parameter or activity (what is intended to happen),
- the guide word (what type of deviation occurs),
- the deviation or “scenario” (formulation of the deviation),
- the possible consequences,
- the existing barriers or measures (if any).

The output of the HAZOP technique is a list of hazardous situations and risks linked to specific process steps and deviations. These outputs are subsequently processed using the modified HRC–PFMEA technique, which is used to quantify the identified risks.

### 5.2 Risk analysis techniques

After hazardous situations have been identified using HTA and HAZOP, the identified risks are further quantified by means of HRC–PFMEA, which enables a structured assessment of their potential impacts.

The Human–Robot Collaboration–Process Failure Mode and Effects Analysis (HRC–PFMEA) technique is a modified version of the classical FMEA approach, adapted for risk assessment in the field of collaborative robotics (HRC). Its purpose is to systematically analyse potential failures arising from human–robot collaboration, evaluate their severity and likelihood of occurrence, and subsequently propose measures to minimise them. While traditional FMEA is based on historical data concerning failures of technical components, HRC–PFMEA focuses on process-related and interaction-related risks arising from human and robot behaviour, as well as from their communication links.

The HRC–PFMEA assessment is based on three parameters: Severity, Occurrence, and Detection. This technique was introduced and applied in study (Antonelli and Stadnicka, 2019), in which the authors adapted the standard FMEA scales to suit the specific environment of collaborative assembly processes. The technique therefore makes it possible to assess human errors, communication failures, and human–robot interactions, and represents a practical tool for preventing errors, injuries, and productivity losses in the early stages of HRC system design.

The authors of this article decided to further adapt this originally modified technique.

#### 5.2.1 Scope and modifications of the HRC–PFMEA technique

In this study, HRC–PFMEA is applied in a laboratory setting to validate the work procedure entitled “Assisted Assembly of a Children’s Skateboard.” For this reason, the following modifications were introduced:

1. Detection is not evaluated.

In this study, Detection is not assessed. Within the proposed technique, Detection is introduced as an optional parameter expressing the ability of existing control mechanisms to identify a failure or deviation before a hazardous situation occurs. In a laboratory scenario involving simulated deviations, the evaluation of Detection would create a false sense of precision, and the results would be highly dependent on the specific instrumentation and simulation method used. The Detection criteria (D) are therefore included in the technique, but they are not assessed within the present laboratory case study. The D parameter is considered an optional extension of the technique for future validation under real operating conditions.

2. Severity is evaluated primarily in terms of impact on human safety.

In study (Antonelli and Stadnicka, 2019), the key factors include not only safety, but also operational impacts such as quality, time, and cost. In the present study, these impacts are divided into two components:

1. S_S (Safety Severity) – the severity of the impact on human health and safety.
2. P_S (Process Severity) – the severity of the impact on the process (quality, time, productivity, and cost).

In this technique, impacts on human health and safety are treated as the primary criterion in risk-related decision-making. Operational impacts are introduced as an optional factor, P_S. Although operational impacts are not assessed in the present study, the parameters for evaluating P_S are retained as a basis for future application in industrial settings, where the assessment may be supported by real operational data.

3. Occurrence (O) is defined as an expert estimate of the likelihood of occurrence.

Occurrence is assessed on the basis of expert judgement. The reason for this modification is the lack of historical data from which the frequency or probability of occurrence could be derived for a specific HRC scenario. This limitation is also highlighted by the authors of study [36].

4. Additional attribute.

Another modification of the technique is the introduction of the Cause Source attribute, which classifies the dominant source of the cause as Robot/Human/Communication/Environment/Organization. The main purpose of this modification is to improve clarity and enable categorisation. Although this attribute does not affect the calculation of the RPN, it provides a clearer overview of risks according to the source of the cause.

#### 5.2.2 Definition of parameters and calculation

##### 5.2.2.1 Detection (D): optional

This factor expresses the ability of existing control mechanisms to detect a failure or deviation before a hazardous situation occurs. D is defined on an ordinal scale from 1 to 10, where 1 corresponds to nearly certain automatic detection and prevention (fail-safe), and 10 corresponds to a failure that is practically undetectable (see Table 2). In the present laboratory case study, D is not quantified (D = N/A).

**TABLE 2:** Criteria of detection.

Description of detectability	Level of detection
Almost certain detection + automatic prevention before a dangerous situation arises (fail-safe)	1
Very high detection. Automatic response, minimal chance of detection failure	2
High detection. Automatic detection, but there are edge scenarios where detection fails	3
Rather high. Detection is automatic, but less sensitive (depends on conditions)	4
Medium. Detection exists but is partially human-dependent or not always active	5
Rather low. Detection is indirect, delayed, or easily circumvented	6
Low. Often detected only after a near-miss or after a dangerous situation has arisen	7
Very low. Detection is more likely to occur after an incident or damage	8
Almost undetectable, not normally detected before an incident or damage occurs. Virtually no effective pre-contact control	9
Undetectable. No mechanism to detect failure in time	10

Rules for assigning D:

D is assessed with respect to the existing measures/barriers and their quality.

1. Is the measure automatic or merely organisational? (automatic → lower D)
2. Is it fail-safe? (yes → significantly lower D)
3. Does the measure detect the hazardous situation before it leads to harm, or only afterwards? (before the adverse consequence → lower D)
4. Is the measure easily bypassed? (easily bypassed → higher D)
5. What is the level of coverage and robustness (e.g., zone coverage, timing, edge cases)? (more robust → lower D)

##### 5.2.2.2 Safety severity (S_S)

This factor reflects the severity of the impact of a hazardous situation on human health and safety. The S_S value is determined with regard to the type of contact (e.g., impact, pinching, entanglement, and others), the energy or motion involved, and the body parts potentially exposed during the interaction.

Real-world data from injury analyses conducted by OSHA indicate that severe finger injuries, including amputations, are common in incidents involving stationary robots, with pinched/pinned mechanisms among the most frequently reported (Sanders et al., 2024). Therefore, these findings are taken into account in Table 3.

**TABLE 3:** Criteria of safety severity.

Description of severity and consequences	Level of severity
Only unpleasant contact and startling of the operator No health consequences	1
Only minor injuries, light abrasions, light skin injuries. Injuries not requiring treatment. No incapacity for work No health consequences	2–3
Mild injuries such as cuts, bruises and injuries requiring treatment. Possible short-term incapacity for work No health consequences	4
Injuries such as deep cuts, light burns, impacts or crushing by the robot, and injuries requiring medical treatment. Short-term incapacity for work No lasting health effects	5–6
Moderate injuries such as fractures, deep cuts, more severe burns. Injuries requiring medical treatment. Possible long-term incapacity for work No permanent health consequences If the specified energy/force limit value for individual body parts according to ISO 10218-2:2025 (Table M4) or also according to ISO/TS 15066:2026 (Table A4) is exceeded	7
Severe injuries such as severe fractures, deep wounds, severe pressure ulcers caused by prolonged pressure on a part of the body (squeezing, crushing). Long-term incapacity for work Possible permanent consequences	8
Critical consequences, such as amputations, life-threatening injuries, serious accidents with permanent, serious consequences	9
Fatal consequences, such as damage to health leading to death	10

To establish an objective engineering foundation for assigning Safety Severity (S_S) values, the assessment is quantitatively anchored in measurable biomechanical force and energy thresholds. If the measured or simulated load values for specific human body regions exceed the limit thresholds specified in ISO 10218-2:2025 (Table M4) or ISO/TS 15066:2016 (Table A4), the identified risk is always assigned a minimum severity rating of S_S = 7.

The exact final rating assigned (ranging from 7 to 10) always depends on the specific nature of the identified risk (e.g., quasi-static vs. transient contact, affected body region, and potential for permanent impairment). Assigning S_S ≥ 7 subsequently triggers the mandatory high-severity safety override rule, ensuring that any hazard exceeding biomechanical limits cannot be classified as low risk and remains evaluated at a minimum level of “Increased”.

##### 5.2.2.3 Process severity (P_S): optional

This factor is optional and captures the impact on the process and its outputs, including quality, time, downtime, and costs. It represents a secondary dimension that may be incorporated into the risk analysis and evaluation. Its purpose is to support the prioritisation of implementing risk reduction measures.

In the present laboratory case study, P_S is not quantified (P_S = N/A). The P_S parameters are presented in Table 4.

**TABLE 4:** Criteria of process severity.

Description of impacts on quality, time, downtime, and costs	Level of severity
The impacts are negligible. No rejects, no downtime, at most minor corrections with no impact on the process	1–2
The impacts are minimal. Local rework or short delays, negligible impact on productivity	3–4
The impacts are moderate. Defects in individual units or repeated rework, noticeable impact on work continuity	5–6
The consequences are significant. Workplace downtime, intervention by an adjuster or maintenance technician, multiple units affected, significant impact on time	7–8
The impacts are critical. Long downtime at the workplace, damage to the equipment (robot), a series of rejects or significant costs	9–10

##### 5.2.2.4 Occurrence (O)

This factor expresses the likelihood of a hazardous situation occurring. As robust historical data are currently not available, O is determined on the basis of expert judgement. The Occurrence parameters are presented in Table 5.

**TABLE 5:** Criteria of occurrence.

Description of occurrence	Level of occurrence
Very unlikely. Requires a confluence of multiple events, failures, and abnormal abuse	1
Theoretically possible, but only under exceptional conditions	2
Occasional may occur due to operator deviation or process tolerances	3
Expected in the longer term, but not typical	4
Possible. This is a known scenario in HRC, expected in the life cycle	5
More likely than not. Without action, realistically multiple times in the life cycle	6
Probable. without action, can be expected repeatedly	7
Repeatedly in the short term. Realistic near-miss scenario	8
Very common. Almost always an opportunity, a small deviation is enough	9
Frequent	10

##### 5.2.2.5 Risk priority number calculation

The full form of the index is RPN = S_S × O × D; when Detection is not evaluated (as in this laboratory scenario), the index reduces to RPN = S_S × O.

- Full form: RPN = S_S × O × D (range 1–1000).
- Short form: RPN = S_S × O (range 1–100).

Due to the laboratory conditions, the abbreviated form of the equation is applied in the following case study.

##### 5.2.2.6 Cause source

Cause Source is categorized of a risk cause and to support the targeting of mitigation measures; it is not intended as a substitute for the Occurrence parameter.

Recommended categories:

- Robot* (hardware/software, trajectory, unexpected activation, EOAT (end effector), etc.)

* The cause originates within the robotic subsystem (robot + EOAT + its control system), for example, a mechanical or electrical failure, incorrect configuration, program error, improperly set limits, or unexpected motion.

- Human (incorrect action, inattention, work ergonomics, etc.)
- Communication** (unclear signal, poor timing, HMI (Human-Machine Interface), shared understanding of system status, etc.)

** The cause lies in the interface or coordination layer (robot ↔ human, robot ↔ PLC/HMI), typically involving poor signal timing, ambiguous signalling, incorrect interpretation of status (e.g., “ready”), signal delay, or signal loss.

- Environment/Organization (layout, obstacles, lighting, work pace, instructions, maintenance mode, etc.)

### 5.3 Risk evaluation technique

A risk matrix is a tool used in risk evaluation that enables a visual and systematic assessment of risk levels based on the combination of two or more factors.

Each assessed risk is represented in the matrix by the coordinates of these parameters, which are multiplied to obtain the so-called Risk Priority Number (RPN), representing the level of the given risk. The result is a clear and structured table that classifies risks into the categories of acceptable risk, conditionally acceptable risk, and unacceptable risk. The risk evaluation parameters are presented in Table 6.

**TABLE 6:** Risk matrix values.

Evaluation	Risk level	Measures
Acceptable	1–2	No action required. Acceptable risk
Low	3–9	The risk remains acceptable, but it is necessary to maintain control
Moderate	10–25	It is recommended that you perform a check
Increased	26–49	It is necessary to conduct an inspection, implement additional measures, and reduce the risk
High	50–79	The risk is high. A risk assessment must be conducted to mitigate the risk
Unacceptable	80–100	The risk is unacceptable. Operations must be halted immediately and a new risk assessment conducted

If the value of the parameter S_S ≥ 7, the minimum measures as for “Increased” always apply.

However, to ensure maximum operator safety and health protection, an exceptional safety rule must be incorporated into the risk evaluation process: if the S_S value is equal to or higher than 7, the risk must always be classified as at least “Increased”, regardless of the calculated RPN value. This rule is critical to prevent the underestimation and insufficient mitigation of severe risks. Without this exception, a dangerous scenario could arise where, with high severity (S_S ≥7 ) and minimal estimated occurrence (O = 1), the resulting RPN would be 7. In a conventional matrix, such a score would fall into the low/acceptable risk category, meaning that a situation posing a serious threat to the operator’s health and safety would remain without mandatory verification of safety measures.

The risk matrix makes it possible to establish priorities for the design and implementation of safety measures. Risks with the highest combination of severity and occurrence are classified as unacceptable and require immediate intervention, whereas low-risk items may be accepted without additional modifications.

### 5.4 Outputs of the proposed technique

Table 7 contains a list of the techniques used, their intended use and expected outputs. For some outputs, possible practical uses beyond risk assessment are suggested.

**TABLE 7:** Outputs of the proposed technique.

Technique	Purpose	Expected output
HTA	Support for risk identification. Systematic division of work processes and operator and robot activities into individual tasks and steps. Identification of key interaction points and critical steps	Hierarchical task structure, HTA plan, list of risk areas and inference points for a given collaborative application
HAZOP	Expanding the identification of critical areas (from HTA) related to specific process steps and specific deviations suitable for risk analysis using the modified HRC - PFMEA technique	List of deviations causes and consequences, hazardous situations and risks of the collaborative application under assessment. Input data for risk analysis
HRC–PFMEA	Quantification of identified hazardous situations and risks in terms of their severity, impact on safety and health, and probability of occurrence. Calculation of the risk index	Extended list of identified risks with severity (S_S), occurrence (O), risk priority number (RPN), source of cause and proposed measures. Input data for risk evaluation
Risk matrix	Risk evaluation is based on a combination of severity and occurrence parameters. Determining the level of risk and prioritizing measures	Visualization of risk levels in a matrix (acceptable, low, moderate, increased, high, unacceptable)
Output from HTA + analysis	The results of the identification and analysis can provide information for setting safety measures and workplace layout. Also, safe work procedures, preparation of training materials for operatorsetc.	Design of technical measures, safe workflow for a specific collaborative application, training materials, internal guidelines

## 6 Case study assisted assembly of children’s skateboard (laboratory scenario)

This case study does not concern a standard industrial assembly process. Rather, it demonstrates the application of the proposed hybrid risk assessment technique to a collaborative application in a laboratory scenario involving the assisted assembly of a children’s skateboard. The scenario includes repetitive handling steps, a shared human–robot workspace, part transfer between the robot and the operator, and a pressing operation. It was designed as a demonstrative and test-oriented human–robot collaboration task and was developed solely as a digital simulation in the RobotStudio® environment, with the aim of verifying the usability, transparency, and methodological continuity of the individual techniques within the proposed hybrid approach (HTA → HAZOP → modified HRC–PFMEA → risk matrix).

The risk assessment carried out in this case study is not intended to be complete; rather, it serves to illustrate the overall logic and to visualise the application of the proposed hybrid technique.

### 6.1 Details

#### 6.1.1 Workplace and product description

The case study is conducted using an ABB GoFa CRB 15000 robot with a maximum payload capacity of 10 kg. The robot is installed on a workbench, part of which is designated as the operator’s workspace. The workbench is mobile, equipped with wheels, and can be stabilized using rubber feet; however, within the proposed application, it is considered a stationary workstation. The robot is equipped with a purpose-designed jaw gripper intended for the manipulation of specific components used in the collaborative application.

The assembled product is a children’s skateboard made of plastic. The assembly task consists of assembling the wheels and attaching them to the skateboard axles. The process involves pressing bearings into plastic wheels and then assembling the wheels onto the skateboard body subassembly, which consists of a plastic deck and axles. The weight of the assembled pressed wheel is 60 g.

**FIGURE 4:**
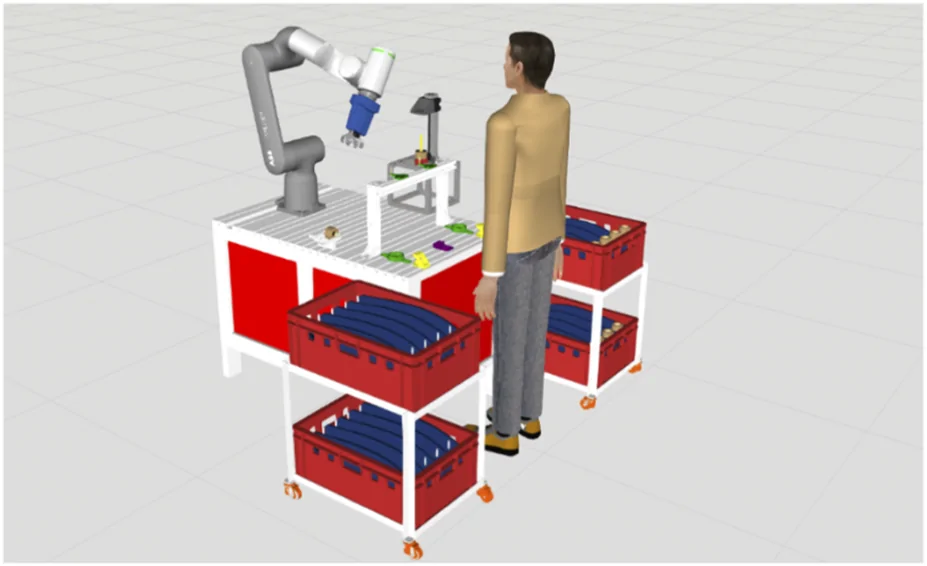
Workstation in simulation.

**FIGURE 5:**
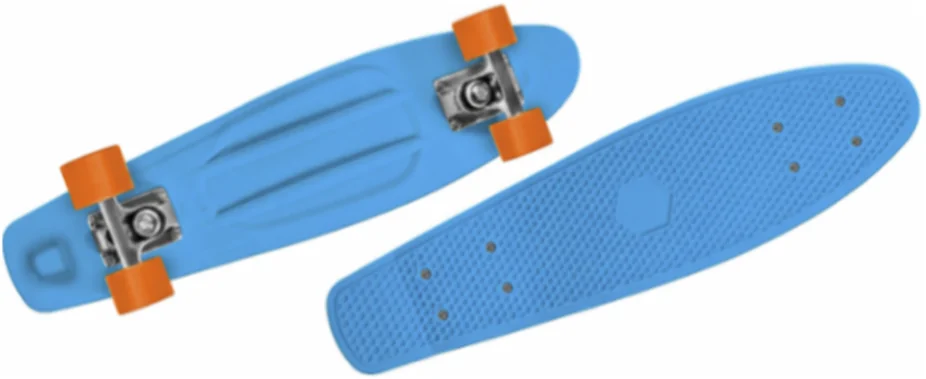
Assembled item.

The workstation is shown in Figure 4, and the assembled object (children’s skateboard) is shown in Figure 5.

#### 6.1.2 Process description (1 cycle: 1 body +4 wheels)

**FIGURE 6:**
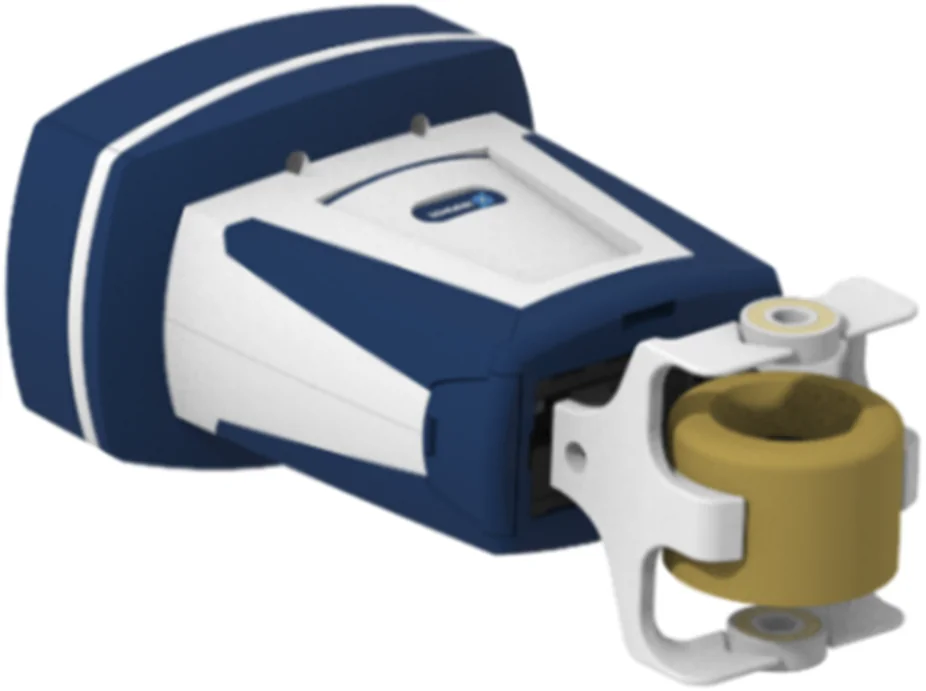
Proposed end-effector prototype.

The process begins with the placement of the skateboard body into a fixture, where it is secured in the assembly position. The robot then picks up the components (two bearings and one plastic wheel) using the designed end-effector, which allows both bearings and the wheel to be grasped simultaneously (see Figure 6). The robot transfers this assembly to the press, where the pressing operation is carried out upon operator confirmation. It subsequently removes the pressed wheel, transfers it to the pick-up position for the operator, and returns to its parking position.

While the robot is moving, the operator simultaneously takes the wheel from the pick-up position, mounts it onto the skateboard axle, secures it with a nut, tightens it, and checks the wheel rotation. In the meantime, the robot repeats the sequence of picking up components and transferring the assembly to the press. After the wheel assembly on one side has been completed, the operator rotates the skateboard body, clamps it into the second assembly position, and repeats the procedure for the opposite side. Once the assembly is completed, the operator removes the finished skateboard from the fixture and places it into the output bin.

#### 6.1.3 Current and future state of the demonstrative collaborative application

Current status:

- Each pick-up position for a wheel has a capacity of one piece.
- After placing the wheel, the robot moves to its parking position.

Continuation of the cycle is controlled via the “confirm/next” button.

- The pressing operation is activated by the operator via the “confirm/next” button.
- In the simulation, the press is considered an open press, that is, without a physical guard.
- At present, no safety measures have been implemented for the collaborative application in the simulation.
- In its current state, the collaborative application is considered only as a simulation and not as a complete physical workstation.

Future status:

- Addition of a wheel presence sensor at the pick-up position.
- Verification of the safe state of the zone by means of safety devices.
- Automation of cycle continuation and, where applicable, activation of the pressing operation.
- Implementation in a real operating environment.

#### 6.1.4 Assumptions and system boundaries

The assessment includes:

- The assisted assembly process of a children’s skateboard (one cycle: one body and four wheels).
- The robotic subsystem, including the end-effector, positioning pin, pick-up position, and pressing operation.
- Operator tasks related to clamping the skateboard, removing the wheels, assembly, tightening, and checking wheel rotation.

Outside the scope of the assessment:

- Replenishment of component supplies (bearings/wheels/rollers).
- Logistics outside the workstation (material supply and removal of containers).
- Maintenance and servicing.
- Quantitative frequency estimates based on long-term statistical data.

The scenario is designed as a demonstrative laboratory application and, at its current stage, is implemented as a simulation without mitigation measures in place.

As no robust historical data are available for the Occurrence parameter, it is determined on the basis of expert judgement.

In this case study, impacts on humans are assessed using S_S, likelihood is assessed using O, and the resulting risk priority is determined as RPN = S_S × O.

The Cause Source attribute is used to classify risks according to the origin of the cause.

The parameters D (Detection) and P_S (Process Severity) are defined in the technique as extensions; however, they are not quantified in the laboratory demonstration.

The workflow of the collaborative application is carried out through the following assembly steps:

- The robot prepares the wheel subassembly by picking up the bearings, the spacer roller, and the plastic wheel, assembling them onto the positioning mandrel, and performing the pressing operation. It then transfers the completed wheel to the pick-up position.
- The operator takes the wheel, mounts it onto the skateboard axle, tightens the nut, and checks the wheel rotation.
- After placing the wheel, the robot moves to a safe parking position and waits for the next command.
- In the current laboratory configuration, the pressing operation is performed using an open press without a physical guard. In the present mode, activation of the pressing operation is carried out by the operator via the “confirm/next” button.

With a view to future implementation in a real operating environment, a variant involving automatic activation of the pressing operation and continuation of the cycle is also discussed. This concept is based on the integration of a camera system to detect and monitor human presence in the relevant zone, supplemented by a sensor for wheel presence detection at the pick-up position and, for example, by guarding of the press.

Within this demonstrative collaborative application, emphasis is placed on the safety aspect of the interaction and on the assessment of impacts on humans. Severity is therefore evaluated using the S_S (Safety Severity) parameter, while the likelihood of occurrence is assessed using the O parameter (expert-judgement likelihood). The risk level is determined as RPN = S_S × O. The Cause Source attribute is used to classify risks according to the origin of the cause. The detection parameter D and the operational impact parameter P_S are not evaluated in this case study.

**FIGURE 7:**
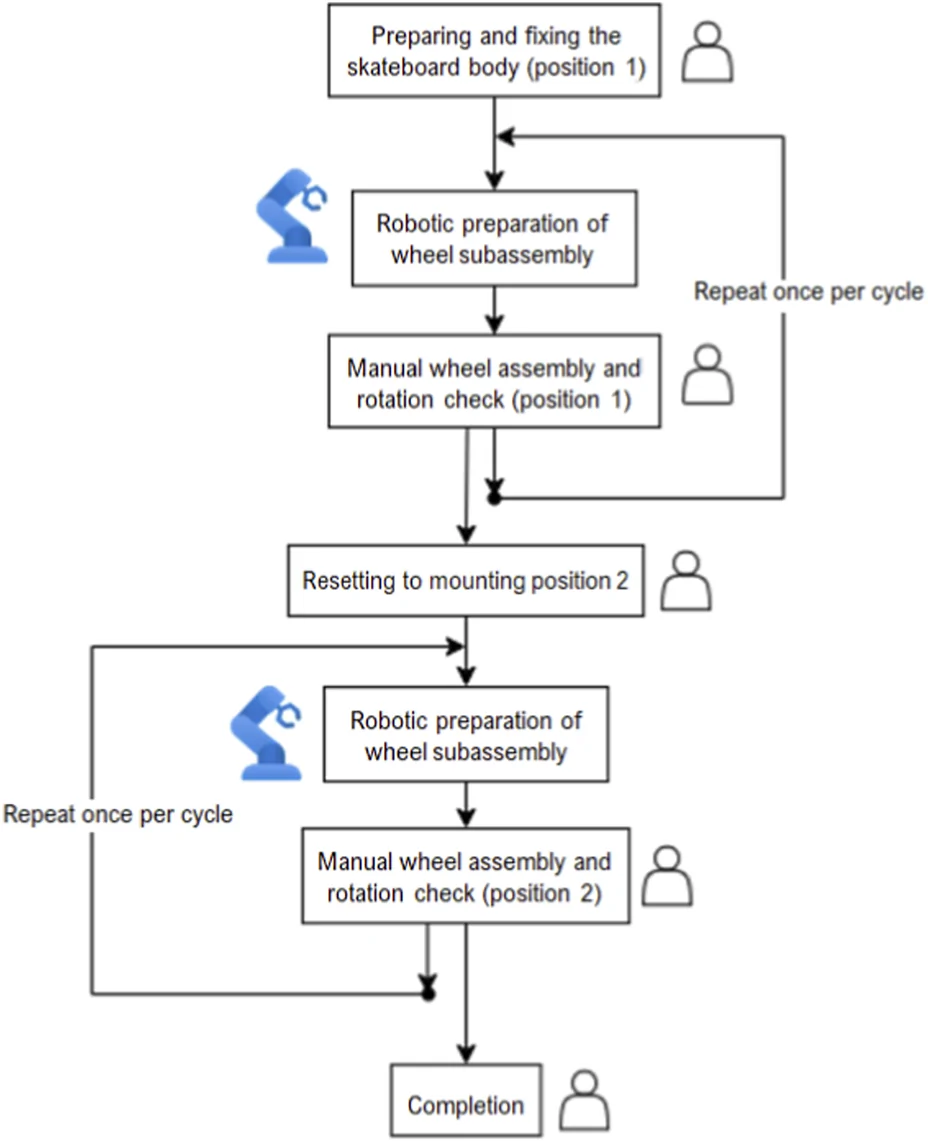
Diagram of the assembly process for a children’s skateboard.

Based on the task decomposition using HTA, selected critical steps of the work process are subsequently chosen to demonstrate the application of the proposed hybrid technique. HAZOP is then applied to these steps in order to identify deviations, their causes, and their possible consequences (the overall scheme is shown in Figure 7). The identified scenarios are transferred into a modified HRC–PFMEA table and evaluated using the RPN. To ensure transparency, all items are labelled in a way that makes it possible to trace the link between the HTA step, the HAZOP deviation, and the corresponding HRC–PFMEA row.

### 6.2 Application of the proposed hybrid technique

#### 6.2.1 Risk assessment procedure

##### 6.2.1.1 Risk identification

HTA.

Level 0:

- Assisted Assembly of a Children’s Skateboard–Completion of Four Wheels and Placement of the Finished Product into the Output Container

Level 1:

1. Preparation and securing of the skateboard body in assembly position 1.
2. Robotic preparation and pressing of the wheel assembly, followed by transfer of the wheel to the pick-up position.
3. Manual wheel assembly, tightening, and rotation check in position 1.
4. Prepositioning the skateboard body (rotation and clamping) to assembly position 2.
5. Robotic preparation and pressing of the wheel assembly, followed by transfer of the wheel to the pick-up position.
6. Manual wheel assembly, tightening, and rotation check in position 2.
7. Removal of the finished skateboard and placement into the output container (completion).

Level 2:

1. Preparation and securing of the skateboard body (position 1).
  1.1. The operator places the skateboard body into the skateboard holder.
  1.2. The operator checks that the skateboard body is properly secured in assembly position 1.

Plan: Steps 1.1–1.2 are performed once per cycle, in the specified order, by the operator.

2. Robotic preparation of the wheel subassembly (one wheel) – performed twice.
  2.1. The robot picks up the wheel assembly (two bearings and one wheel) from the pick-up position or from the magazine.
  2.2. The robot transfers the wheel assembly to the press.
  2.3. The operator initiates the pressing operation (activated via a button).
  2.4. The robot removes the pressed wheel.
  2.5. The robot places the wheel at the pick-up position for the operator.
  2.6. The robot moves to the parking position and waits until the wheel is removed (the operator confirms wheel removal via a button).
  2.7. Start of the next wheel preparation cycle.

Plan: Steps 2.1–2.6 are performed twice in succession (for a total of two wheels). Steps 2.1–2.7 are performed in the specified order, by the robot and the operator.

3. Manual wheel assembly and rotation check (skateboard body in position 1).
  3.1. The operator removes the wheel from the pick-up position.
  3.2. The operator mounts the wheel onto the skateboard axle.
  3.3. The operator secures it with a nut and tightens it.
  3.4. The operator secures the wheel rotation.

Plan: Step 3.1 may only take place after step 2.6 and before step 2.7. Steps 3.1–3.4 are repeated for the next wheel (a total of two times), in the specified order, by the operator.

4. Prepositioning the skateboard body into assembly position 2.
  4.1. The operator rotates the skateboard body.
  4.2. The operator clamps the skateboard body into assembly position 2.

Plan: Steps 4.1–4.2 are performed once per cycle, in the specified order, by the operator.

5. Robotic preparation of the wheel subassembly (one wheel) – performed twice.
  5.1. The robot picks up the wheel assembly (two bearings and one wheel) from the pick-up position or from the magazine.
  5.2. The robot transfers the wheel assembly to the press.
  5.3. The operator initiates the pressing operation (activated via a button).
  5.4. The robot removes the pressed wheel.
  5.5. The robot places the wheel at the pick-up position for the operator.
  5.6. The robot moves to the parking position and waits until the wheel is removed (the operator confirms wheel removal via a button).
  5.7. Start of the next wheel preparation cycle.

Plan: Steps 5.1–5.6 are performed twice in succession (for a total of two wheels). Steps 5.1–5.7 are performed in the specified order, by the robot and the operator.

6. Manual wheel assembly and rotation check (skateboard body in position 2).
  6.1. The operator removes the wheel from the pick-up position.
  6.2. The operator mounts the wheel onto the skateboard axle.
  6.3. The operator secures it with a nut and tightens it.
  6.4. The operator secures the wheel rotation.

Plan: Step 6.1 may only take place after step 5.6 and before step 5.7. Steps 6.1–6.4 are repeated for the next wheel (a total of two times), in the specified order, by the operator.

7. Completion.
  7.1. The operator removes the finished skateboard from the holder.
  7.2. The operator places the finished skateboard into the output container.
  7.3. The operator places the next skateboard body into the holder (position 1) and begins the next assembly cycle (see steps 1.1–7.2).

Plan: Steps 7.1–7.3 are performed in the specified order, by the operator.

HAZOP.

Table 8 defines the groups of risks that can be identified within the collaborative application. Not all risks are identified within the scope of this study. Table 9 contains the use of key words at selected critical points. The use of the HAZOP technique is for illustrative purposes only. The identification of risks is not exhaustive.

**TABLE 8:** Risk distribution by group.

Sig	Type of hazard	Origin	Possible cause	Possible consequences
I	Mechanical	Only some risks demonstrate the use of the technique		
II.	Electrical	A complete risk identification was not performed in this case study. The listed hazard categories will be assigned to specific identified risks in future research		
III.	Thermal			
IV.	Noise			
V	Vibration			
VI.	Material			
VII.	Ergonomic			
VIII.	Environmental			
IX.	Cyber			
X	Combinations			

**TABLE 9:** Application of HAZOP technique.

HTA step	Sig	Type of hazard	Activity	Guide words	Deviation	Possible cause	Potential consequences
2.3., 5.3	I	Mechanical	Press/pressing	None	The press will not start	Signal failure or error	Primarily a process-related impact, with possible operator improvisation
				Early	The press starts too early	Incorrect timing or unclear “ready” status	Collision or damage, with a risk of pinching during corrective intervention in the press area
				In addition	Repeating pressing cycle	Signal failure or error	Unexpected press movement that may result in upper-limb pinching during corrective intervention or in entrapment of the robot end-effector
				Other than	Pressing a misaligned part	Inaccurate placement or displacement of the part	Risk of upper-limb pinching (hand or fingers) during manual correction of part placement
2.2., 2.5, 5.2, 5.5			Wheel storage	Other than	The wheel is installed incorrectly	Inaccurate placement or displacement of the part	Risk of collision or hand injury
3.1., 6.1			Removing the wheel	Before	The operator enters the area before the wheel has been fully placed in the pick-up position	Operator error	Risk of finger or hand pinching for the operator
					The operator enters the area before the robot comes to a complete stop (parking position)	Operator error	Risk of collision with the operator’s upper limb
				Other than	The operator uses another hand or both hands to remove the wheel	Operator error	Risk of collision with the operator’s upper limb

#### 6.2.2 Risk analysis and evaluation

The next step is to carry out the risk analysis and risk evaluation. Risk analysis is performed using the HRC–PFMEA technique to quantify the identified hazardous scenarios and risks. Risk evaluation is then conducted by means of the defined risk matrix.

The colour coding of the RPN is selected according to the corresponding risk level (low, medium, high) based on the calculated values. This step can be seen in Table 10. The qualitative scoring of the parameters (S_S and O) presented in Table 10 was conducted by the article’s author team. The evaluation group consisted of six co-authors from VSB–Technical University of Ostrava, representing key multidisciplinary fields essential for a comprehensive safety assessment of human-robot collaboration (HRC). Specifically, Daniel Hartmann and Ales Vysocky (Faculty of Mechanical Engineering) provided expertise in machinery mechanization and robotic system design; Kristyna Hamrikova and Vendula Laciok (Faculty of Safety Engineering) focused on machinery and process safety; Ales Bernatik (Faculty of Safety Engineering) contributed expertise in process safety management; and Radim Hercik (Faculty of Electrical Engineering and Computer Science) addressed functional safety. The values ​​were determined using a structured brainstorming technique. Each identified risk scenario was evaluated iteratively, and parameter values ​​were established based on the authors’ combined expertise, diverse professional backgrounds, and experience, with a minimum consensus of at least five evaluators required for all assigned values.

**TABLE 10:** Using HRC-PFMEA and Risk matrix techniques.

Risk identification				Existing measures	Risk analysis and evaluation				New measures	Repeated risk analysis and evaluation		
ID	Step HTA	Identified hazardous scenario	Risk		S_S	O	RPN	Cause source	Proposed measures	S_S	O	RPN
1	2.3., 5.3	Unexpected activation of the press	Collision/pinching/hand contusion	The press is located farther from the operator, outside the operator’s standard workspace and activation of the operation requires confirmation via the “confirm/next” button	5	5	25	Communication	Physical barrier And safe cycle enable And Repeated start prevention	5	2	10
2	2.5., 5.5	The wheel is improperly positioned	Collision/hand injury	Visual check of placement	4	8	32	Environment	Precise guidance of the wheel into the pick-up position And Finger-safe edges And Detection of correct wheel seating And Camera monitoring	6	2	12
3		Premature start of the next wheel assembly cycle (“confirm” pressed too early)	Impact/collision/pinching/hand contusion	Safe robot position (waiting for operator confirmation). Operator confirmation of the action via “confirm/next”	6	7	42	Communication	Wheel presence sensor And Safety device for monitoring a clear zone	6	1	6
4	3.1., 6.1	The operator enters the area before the wheel has been fully placed onto the skateboard axle	Finger or hand pinching	Currently, no measures re in place	5	9	45	Presumed human error	Camera-based detection of the operator’s upper limbs And Setting of force limits (safe values in accordance with ISO 10218:2025)	3	2	6
5			Finger or hand puncture injury	Currently, no measures are in place	7	9	63	Presumed human error	Camera-based detection of the operator’s upper limbs And Setting of force limits (safe values in accordance with ISO 10218:2025)	3	2	6

## 7 Discussion

The application of the proposed integrated hybrid technique to the simulation of assisted skateboard assembly demonstrated a practical workflow and methodological continuity between task decomposition, deviation identification, and risk assessment. It is important to emphasize that this assessment represents a risk assessment conducted in the early design phase of the collaborative application. As a result, the initial baseline scenario was intentionally evaluated in an unmitigated state–without prior physical or functional safety barriers. The primary goal of applying the proposed hybrid technique at this conceptual stage is precisely to systematically identify inherent process risks and define the necessary technical, physical, and organizational safety measures prior to the physical construction and deployment of the workstation.

**FIGURE 8:**
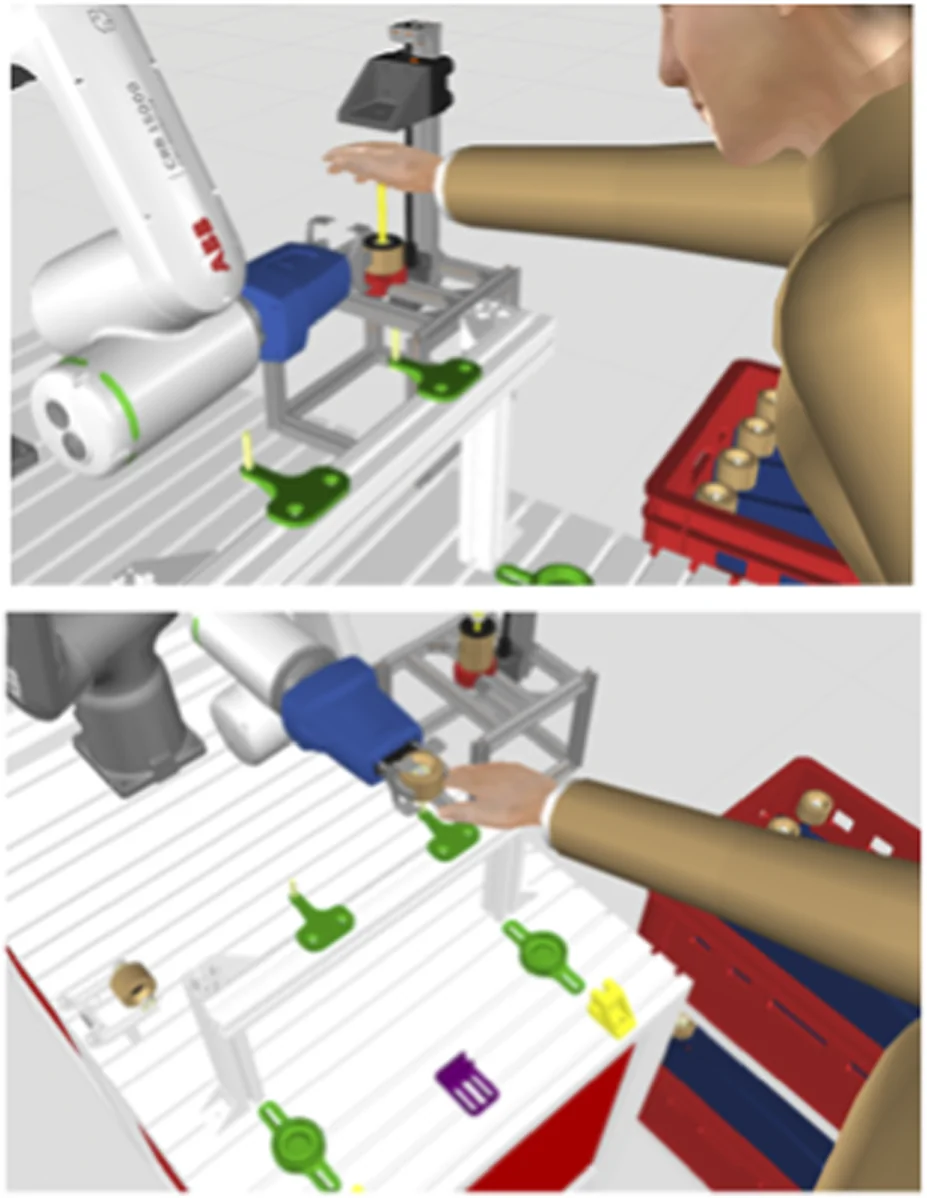
Dangerous situation when pressing and removing the wheel.

The initial risk analysis revealed that the most critical hazardous scenarios were associated with manual interaction during robotic handling and pressing operations (specifically IDs 3, 4, and 5 in Table 10). In this unmitigated baseline, the highest risk priority numbers were due to the potential for serious injury, such as pinched fingers or hands, and stab wounds during premature operator entry into a shared workspace or open press area shown in Figure 8. These findings closely align with real-world accident statistics reported by OSHA (Sanders et al., 2024), where upper extremity injuries, contusions, and finger amputations are the predominant hazards in stationary robotic applications. By identifying these critical points early in the design process, targeted safety measures–such as camera-based upper extremity detection, physical barriers, and force and speed limitations per ISO 10218-2:2025 (International Organization for Standardization, 2025a) – were formally defined and integrated into the evolving system design, thereby reducing the level of risk.

Once effective engineering measures are defined and implemented, the probability of occurrence (O) is significantly reduced, mitigating the risk without having to rely solely on operator vigilance. Furthermore, in accordance with the requirements of ISO 10218-2:2025 (International Organization for Standardization, 2025a), ISO/TS 15066:2016 (Table A4) (International Organization of Standardization, 2016), and binding legal frameworks such as Regulation (EU) 2023/1230 on Machinery (Regulation, 2023), serious health consequences must be addressed by mandatory technical protective measures regardless of their estimated occurrence. Relying solely on low estimated frequency to justify high severity risks creates a false sense of security. The proposed hybrid framework enforces this principle by incorporating a high-severity safety override rule S_S ≥ 7, ensuring that hazards exceeding biomechanical energy limits automatically trigger physical or functional safety interventions and cannot be classified as low risk without mandatory verification of the protective barriers.

The practical benefit of the proposed technique lies in the early-stage elimination of risks. Although the assessment was conducted in a simulation environment, this initial virtual design provides significant economic benefits. Identifying and mitigating risks directly within the simulation substantially reduces the financial costs of additional structural modifications that would otherwise be inevitable during the physical installation and commissioning of the machine or production line. The outputs from HTA and HAZOP serve directly as a basis for the creation of standard operating procedures (SOP) and operator training. The connection of HTA steps, HAZOP deviations, HRC–PFMEA, and protective measures allows for the traceability required for CE conformity assessment according to ISO 10218-2:2025 and applicable national OHS legislation. In addition, targeted risk identification prevents unnecessary oversizing of safety features and maintains the speed of the production cycle.

The study successfully verified the logical structure and traceability of the technique, but a key limitation remains the reliance on expert consensus for the occurrence (O) parameter under simulated conditions. Simulation environments cannot fully replicate unpredictable operator behavior, fine mechanical tolerances, or transient contact dynamics. To bridge the gap between simulation-based estimates and reality, ongoing research is focused on building a physical workplace for this collaborative application.

## 8 Conclusion

This article introduced a new integrated hybrid risk assessment technique designed specifically designed for human-robot collaboration (HRC), which systematically combines hierarchical task analysis (HTA), hazard and operability study (HAZOP), process failure mode and effects analysis adapted for HRC processes (HRC–PFMEA), and risk matrix. This integrated framework enables structured process decomposition, formulation of deviation scenarios, qualitative and quantitative risk assessment, and decision-making on risk reduction or elimination measures in the early stages of system design. The main limitation of this study is that the proposed technique was validated exclusively in a digital simulation environment using a demonstration workstation for assisted skateboard assembly. Although the simulation successfully demonstrated the methodological procedure, traceability, and logical sequence of analytical steps, consensus-based expert assessment under simulated conditions cannot fully replace operational and empirical data. Given these limitations, ongoing research focuses on building a physical workplace for this collaborative application that will allow direct empirical measurement of dynamic contacts, their impact forces, and pressures. These measured values ​​will be compared with the biomechanical values ​​specified in ISO/TS 15066:2016 and ISO 10218-2:2025, allowing the multidisciplinary team to quantitatively refine the safety severity scale (S_S) and replace expert estimates with empirically validated data.

## Data Availability

The original contributions presented in the study are included in the article/Supplementary Material, further inquiries can be directed to the corresponding author.
